# Global longitudinal strain improves risk assessment after ST-segment elevation myocardial infarction: a comparative prognostic evaluation of left ventricular functional parameters

**DOI:** 10.1007/s00392-021-01855-6

**Published:** 2021-04-21

**Authors:** Magdalena Holzknecht, Martin Reindl, Christina Tiller, Sebastian J. Reinstadler, Ivan Lechner, Mathias Pamminger, Johannes P. Schwaiger, Gert Klug, Axel Bauer, Bernhard Metzler, Agnes Mayr

**Affiliations:** 1 University Clinic of Internal Medicine III, Cardiology and Angiology, Medical University of Innsbruck, Anichstrasse 35, 6020 Innsbruck, Austria; 2 University Clinic of Radiology, Medical University of Innsbruck, Anichstrasse 35, 6020 Innsbruck, Austria; 3 Department of Internal Medicine, Academic Teaching Hospital Hall in Tirol, Milser Strasse 10, 6060 Hall in Tirol, Austria

**Keywords:** ST-segment elevation myocardial infarction, Cardiac magnetic resonance imaging, Left ventricular function, Myocardial strain, Prognosis

## Abstract

**Aim:**

We aimed to investigate the comparative prognostic value of left ventricular ejection fraction (LVEF), mitral annular plane systolic excursion (MAPSE), fast manual long-axis strain (LAS) and global longitudinal strain (GLS) determined by cardiac magnetic resonance (CMR) in patients after ST-segment elevation myocardial infarction (STEMI).

**Methods and results:**

This observational cohort study included 445 acute STEMI patients treated with primary percutaneous coronary intervention (pPCI). Comprehensive CMR examinations were performed 3 [interquartile range (IQR): 2–4] days after pPCI for the determination of left ventricular (LV) functional parameters and infarct characteristics. Primary endpoint was the occurrence of major adverse cardiac events (MACE) defined as composite of death, re-infarction and congestive heart failure. During a follow-up of 16 [IQR: 12–49] months, 48 (11%) patients experienced a MACE. LVEF (*p* = 0.023), MAPSE (*p* < 0.001), LAS (*p* < 0.001) and GLS (*p* < 0.001) were significantly related to MACE. According to receiver operating characteristic analyses, only the area under the curve (AUC) of GLS was significantly higher compared to LVEF (0.69, 95% confidence interval (CI) 0.64–0.73; *p* < 0.001 vs. 0.60, 95% CI 0.55–0.65; *p* = 0.031. AUC difference: 0.09, *p* = 0.020). After multivariable analysis, GLS emerged as independent predictor of MACE even after adjustment for LV function, infarct size and microvascular obstruction (hazard ratio (HR): 1.13, 95% CI 1.01–1.27; *p* = 0.030), as well as angiographical (HR: 1.13, 95% CI 1.01–1.28; *p* = 0.037) and clinical parameters (HR: 1.16, 95% CI 1.05–1.29; *p* = 0.003).

**Conclusion:**

GLS emerged as independent predictor of MACE after adjustment for parameters of LV function and myocardial damage as well as angiographical and clinical characteristics with superior prognostic validity compared to LVEF.

**Graphic abstract:**

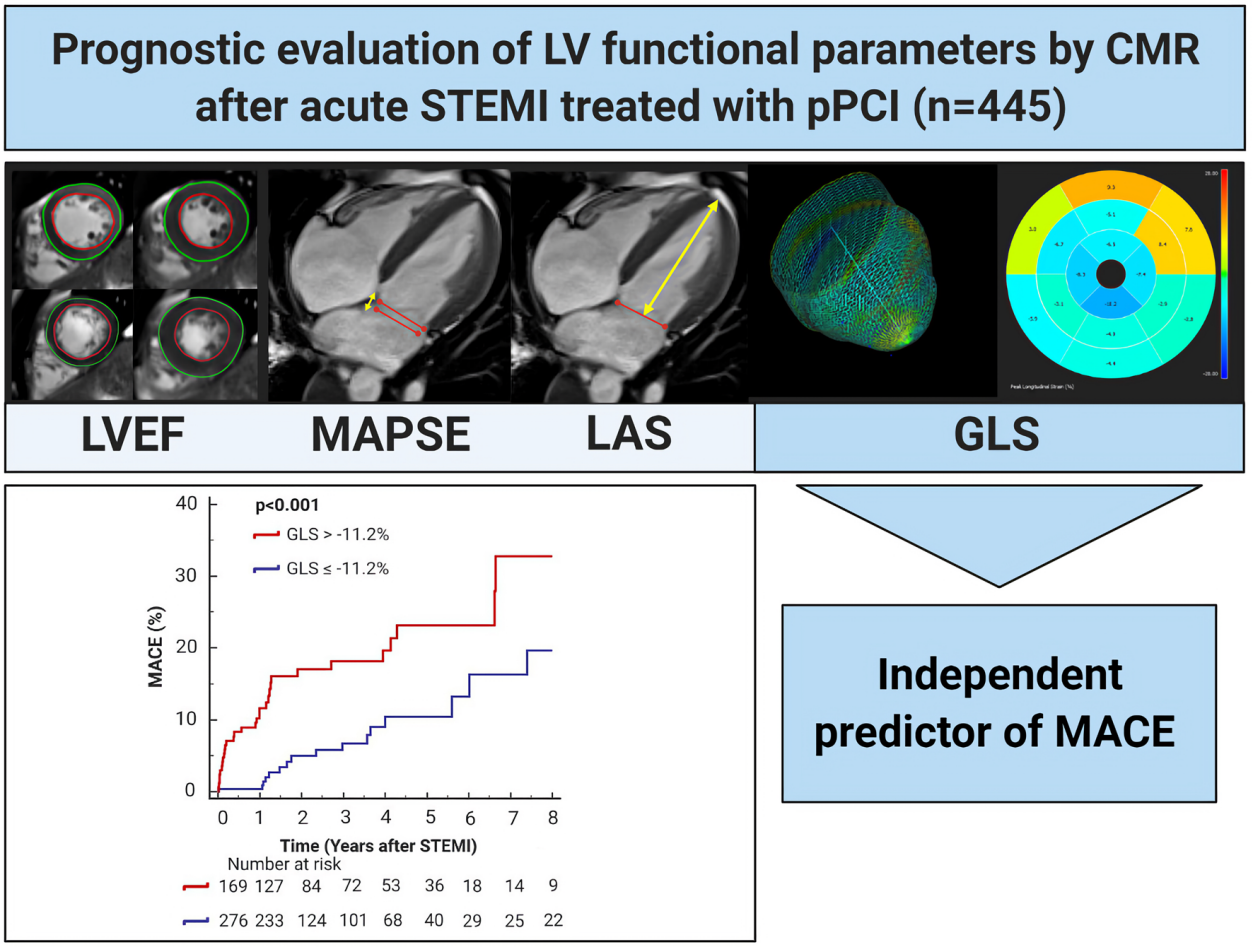

## Introduction

Despite remarkable advances in the management of patients suffering from ST-segment elevation myocardial infarction (STEMI), the risk of subsequent cardiovascular complications and mortality post-STEMI still remains considerable [1]. Therefore, adequate individual risk assessment following revascularization is of crucial clinical importance [2].

Remnant left ventricular (LV) function is a key determinant in decision-making after acute myocardial infarction and has a strong impact on short- and long-term prognosis [2]. LV function parameters are generally determined by echocardiography, which, however, has numerous limitations, such as moderate spatial resolution, considerable inter-observer variabilities and insufficient acoustic windows [3]. Cardiac magnetic resonance (CMR) imaging represents the current gold standard to determine LV volumes and function and is the optimal imaging modality to evaluate subtle changes in the post-infarcted myocardium [4]. LV ejection fraction (LVEF) is a well-established marker of global LV function and recommended for risk prediction after STEMI with important implications for patient management [2, 5]. Nevertheless, LVEF does not allow the assessment of subtle regional differences in cardiac function, hampering the prognostic accuracy of isolated LVEF assessment [6].

Alternative approaches for the determination of LV function with a more subtle depiction of longitudinal shortening in the course of ischemia are mitral annular plane systolic excursion (MAPSE) and fast manual long-axis strain (LAS) [7–9]. MAPSE, displaying atrioventricular plane motion, and LAS, comparing end-diastolic and end-systolic diameters of the LV, are suggested as easy-to-measure surrogate parameters for LV function due to their fast assessment without the need of an additional software [9, 10]. Finally, global longitudinal strain (GLS) permits to detect delicate changes in post-infarcted myocardium enabling a more accurate assessment of systolic function with superior prognostic validity as compared to LVEF [11–13].

In the context of acute STEMI, GLS has been shown to independently predict major adverse cardiac events (MACE) after revascularized STEMI compared with conventional LVEF [14, 15]. Besides, MAPSE is suggested to provide significant higher prognostic validity than LVEF in this patient population [9]. Also LAS has been shown to predict MACE after acute myocardial infarction over established clinical parameters [8]. However, no study compared the prognostic significance of all of these parameters in terms of predicting hard clinical events so far.

We, therefore, aimed to investigate the comparative prognostic value of LVEF, MAPSE, LAS and GLS by CMR in the acute stage post-STEMI for the occurrence of MACE.

## Methods

Prior to study inclusion, written informed consent was given by all participants. The study was designed and conducted in compliance with the Declaration of Helsinki and received approval by the research ethics committee of the Medical University of Innsbruck.

### Patient population and endpoint definitions

For this prospective observational CMR study, patients consecutively enrolled in the “Magnetic Resonance Imaging In Acute ST-Elevation Myocardial Infarction (MARINA-STEMI) trial” (NCT04113356) between 2011 and 2019 were evaluated for inclusion in the final analysis. Inclusion criteria were: first STEMI according to the ESC/ACC/AHA committee criteria [16], revascularization by primary percutaneous coronary intervention (pPCI) within 24 h after onset of ischemic symptoms and Killip class < 3 at time of CMR. The following exclusion criteria were applied: inability or unwilling to sign written informed consent, age < 18 years, any history of previous myocardial infarction or coronary intervention, an estimated glomerular filtration rate < 30 mL/min per 1.73 m^2^ and any other contraindication to CMR examination (pacemaker, severe claustrophobia, orbital foreign body, cerebral aneurysm clip, or known or suggested contrast agent allergy to gadolinium) [17].

Primary endpoint of the study was the occurrence of MACE, including all-cause death, myocardial re-infarction and new congestive heart failure after discharge for the index event. Re-infarction was defined according to the redefined ESC/ACC/AHA committee criteria: symptoms of ischemia and/or new significant ST-segment changes with a rise and/or fall of hs-cTnT with at least one value above the 99th percentile upper reference limit in patients with normalized values or increase of 50% in the setting of non-normalized troponin value [16]. New congestive heart failure was determined as first episode of cardiac decompensation requiring intravenous diuretic therapy with or without hospital re-admission [18]. Clinical endpoints were collected via telephone interviews at 6 months, 12 months and then every 12 months thereafter, using a standardized questionnaire. Follow-up investigations were carried out by personnel blinded to all baseline data which checked the declared endpoints afterwards by reviewing the medical records.

### Cardiac magnetic resonance imaging

All patients were investigated in supine position on a 1.5 T clinical MR scanner (MAGNETOM Avanto^fit^; Siemens Healthineers AG, Erlangen, Germany) within the first week after treatment with pPCI using an 18-channel body phased-array surface coil together with the integrated 32-channel spine matrix coil. The standardized imaging protocol of our research group was published in detail in previous work [17].

High-resolution cine images in long and short axes covering the LV (10–12 slices) were acquired using a balanced steady-state free precession (bSSFP) sequence with retrospective electrocardiographic (ECG) gating (slice thickness (SL): 8 mm, interslice gap: 2 mm, echo time (TE) 1.19 ms, repetition time (TR) 2.83 ms, 22 lines per segments, temporal frame duration 62.26 ms, frame rate 25 frames per second (fps), flip angle (FA) 70°, field of view (FoV) 380 × 310 mm, matrix 320 × 260, voxel size (VS) 2.6 × 1.8 × 8.0 mm^3^) parallel imaging mode: GRAPPA (generalized auto-calibrating partial parallel acquisition) with acceleration factor: 2).

**Fig. 1:**
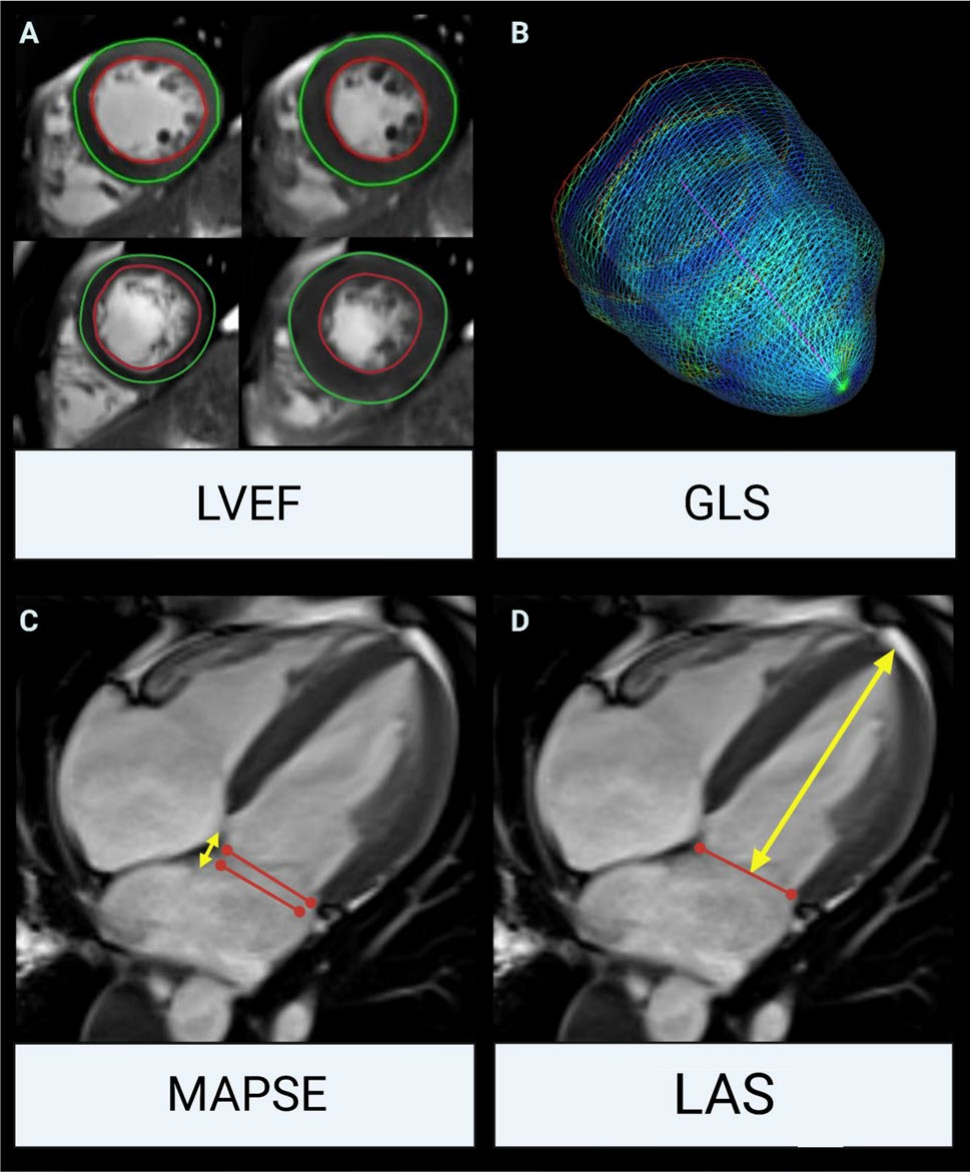
Schematic illustration of LV functional parameters by CMR. **a** LVEF assessment on end-systolic and end-diastolic short-axis cine images. **b** Three-dimensional GLS was assessed using feature tracking. **c** MAPSE (end-systolic to end-diastolic plane, yellow arrows) and **d** LAS evaluation on 4-chamber cine views (distance between the origins of the mitral leaflets and the epicardial border of the LV apex, yellow arrows). *LV* left ventricular, *CMR* cardiac magnetic resonance, *LVEF* left ventricular ejection fraction, *GLS* global longitudinal strain, *MAPSE* mitral annular plane systolic excursion, *LAS* fast manual long-axis strain

For the assessment of LV volumes and LVEF on short-axis cine images, a standard post-processing software (ARGUS; Siemens Healthineers AG, Erlangen, Germany) was applied (Fig. 1, Panel A) with semi-automatic detection of LV endo- and epicardial borders.

Feature tracking analyses were performed by one experienced observer using the commercially available LV specific cvi42 Tissue Tracking software (Circle Cardiovascular Imaging Inc®, Calgary, Canada, v5.1.4), as described previously [14]. LV endocardial and epicardial borders were manually traced at end-diastole in short- and long-axis cine images using a point-and-click approach. Short-axis measurements were conducted at predefined apical, mid-ventricular and basal levels and long-axis data were derived by tracing three standardized planes (2-chamber, 3-chamber as well as 4-chamber views). Papillary muscles were assigned to the LV volume. Based on the 16-segment model, the software algorithm calculated 3-dimensional peak strains and subsequently, by averaging the according peak values of the segments, the global strain parameters: GLS (Fig. 1, Panel b), global radial strain (GRS) and global circumferential strain (GCS).

The standardized measurement procedure for MAPSE has been reported previously [9]. Briefly, MAPSE was measured as the perpendicular distance of the end-systolic mitral annular plane to the end-diastolic plane, measured in regard to the septal attachment of the mitral valve in end-diastole on a long-axis 4-chamber cine view (Fig. 1, Panel c).

Intra- and inter-observer variabilities for feature tracking CMR and MAPSE measurements have been published previously [9, 14].

LAS was assessed on a cine 4-chamber view. As described previously [10], the distance between the epicardial border of the LV apex and the middle of a line connecting the origins of the mitral leaflets was measured in end-diastole and end-systole (Fig. 1, Panel D). Thereafter, LAS was calculated with the following formula:

$$\mathrm{LAS}= \frac{{\mathrm{Length}}_{\mathrm{end}-\mathrm{systole}}- {\mathrm{Length}}_{\mathrm{end}-\mathrm{diastole}}}{{\mathrm{Length}}_{\mathrm{end}-\mathrm{diastole}}}*100$$

An ECG-triggered, phase-sensitive inversion recovery sequence was used to obtain late gadolinium enhancement (LGE) images 15–20 min after application of 0.2 mmol/kg of Gd-DO3A-butriol (Gadovist®, Bayer Vital GmbH, Leverkusen, Germany) with short-axis slices covering the entire left ventricle and TI individually adjusted to null signal from normal myocardium (IR bSSFP sequence with phase-sensitive and magnitude reconstructed images; SL: 8 mm, interslice gap: 2 mm, FoV: 400 × 363 mm, matrix: 256 × 232, VS: 2.2 × 1.6 × 8.0 mm^3^, TR: 2.9 ms, TE:1.2 ms, FA: 45°, parallel imaging mode: GRAPPA, acceleration factor: 2). For quantification of infarct size (IS), a picture archiving and communication system (PACS) workstation (IMPAX®, Agfa HealthCare, Bonn, Germany) was used, whereas “hyper-enhancement” was defined as + 5 standard deviations above the signal intensity of remote LV myocardium [18, 19]. IS was expressed as percentage of total LV myocardial mass (LVMM). Microvascular obstruction (MVO) was defined as persisting area of “hypo-enhancement” within the hyper-enhanced territory and was also reported as percentage of LVMM [18]. Regions of MVO were included in aggregate IS.

Experienced observers, blinded to clinical and angiographic data, analyzed all CMR images.

Incomplete CMR was defined as premature termination of the investigation due to patient, medical or technical problems.

### Statistical analyses

Continuous variables were expressed as median with interquartile range (IQR) and categorical variables as numbers with corresponding percentages. Differences in continuous and categorical variables between two groups were tested by Mann–Whitney *U *test and Chi-square test, respectively. Univariable and multivariable Cox regression analyses were performed to reveal predictors of MACE. All variables with a *p* ≤ 0.10 in univariable analysis were included in multivariable testing. To ensure statistical robustness with respect to our sample size and number of events, 3 multivariable Cox regression models (CMR, angiographical and clinical model) were compiled. The CMR model comprises parameters of LV function and myocardial damage, the angiographical model contains independent predictors of the CMR model adjusted for interventional parameters, whereas in the clinical model, the independent LV function parameter of the angiographical model was adjusted for markers of clinical prognosis. Collinearity was evaluated by the variance inflation factor (VIF), whereas a VIF > 5 was interpreted as relevant collinearity, > 10 as serious collinearity [20]. Receiver operating characteristic (ROC) analyses were applied to evaluate area under the curve (AUC) for the prediction of MACE. ROC curves were compared according to DeLong et al. [21]. Following Rice and Harris, AUC values were categorized as negligible (≤ 0.55), small (0.56–0.63), moderate (0.64–0.70) and strong (≥ 0.71) [22]. The incremental prognostic value was determined using reclassification analyses via R package ‘PredictABEL’. Continuous net reclassification improvement (NRI) and integrated discrimination improvement (IDI) were calculated. Youden Index was calculated to evaluate the optimal cut-off values for dichotomization of continuous MACE predictors [23]. MACE-free survival was estimated and depicted by the Kaplan–Meier method and differences were assessed by the log-rank test. All tests were 2-tailed and the significance level was set at 0.05. SPSS Statistics v26.0 (IBM, Armonk, New York), MedCalc v19.0.7 (Ostend, Belgium) and R 3.6.1 (The R Foundation, Austria) were used for statistical analyses. The graphic abstract was created with BioRender.com.

## Results

### Study population

**Fig. 2:**
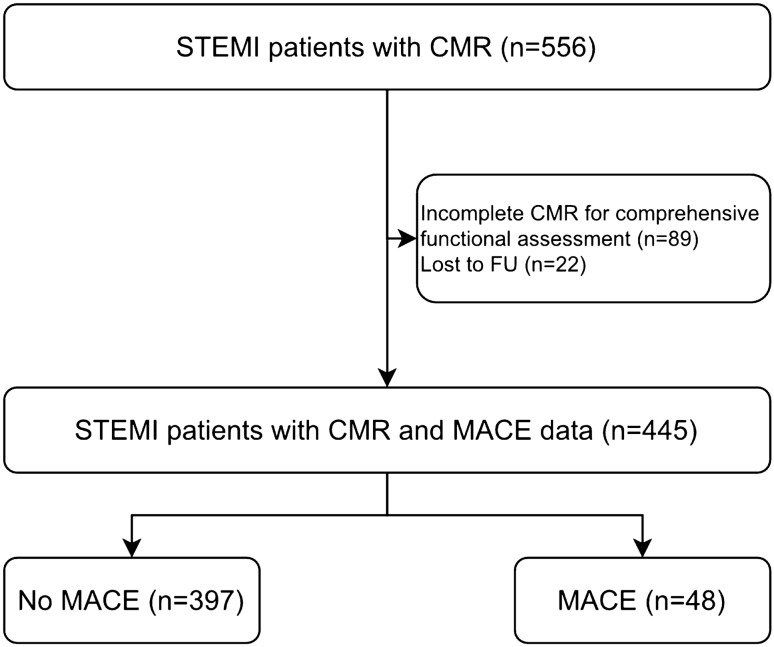
Flow diagram of the study cohort. *STEMI* ST-segment elevation myocardial infarction, *FU* follow-up, *MACE *major adverse cardiac events, *CMR* cardiac magnetic resonance

In total, 445 STEMI patients were included in the final analysis. A detailed study flow chart that includes the reasons for exclusion is depicted in Fig. 2.

Median age of the overall cohort was 57 [IQR: 50–66] years and 67 patients (15%) were female. Total ischemia time was in median 197 [IQR: 126-344] minutes. Baseline patient characteristics are provided in detail in Table 1.

**Table 1:** Patient characteristics

	Total population (*n* = 445)	No MACE (*n* = 397, 89%)	MACE (*n* = 48, 11%)	*p*-value
Age, years	57 [50–66]	57 [49–65]	62 [51–72]	**0.023**
Female, *n* (%)	67 (15)	58 (15)	9 (19)	0.449
Body mass index, kg/m^2^	26.2 [24.6–28.4]	26.2 [24.7–28.6]	25.9 [24.1–27.8]	0.233
Current smoker, *n* (%)	246 (55)	220 (55)	26 (54)	0.869
Hyperlipidemia, *n* (%)	265 (60)	238 (60)	27 (56)	0.622
Diabetes mellitus, *n* (%)	40 (9)	31 (8)	9 (19)	**0.012**
Family history, *n* (%)	140 (32)	127 (32)	13 (27)	0.489
Hypertension, *n* (%)	230 (52)	192 (48)	38 (79)	** < 0.001**
Systolic blood pressure, mmHg	132 [115–152]	132 [115–152]	138 [116–156]	0.677
Diastolic blood pressure, mmHg	80 [71–95]	80 [71–94]	85 [70–99]	0.712
Heart rate, bpm	72 [63–85]	71 [62–85]	74 [63–85]	0.831
Total ischemia time, min	197 [126–344]	195 [124–332]	223 [137–593]	0.146
Door-to-balloon time, min	37 [18–70]	37 [18–69]	44 [19–99]	0.263
Culprit lesion, *n* (%)				0.083
RCA	182 (41)	165 (41)	17 (35)	
LAD	202 (45)	173 (44)	29 (61)	
LCX	58 (13)	56 (14)	2 (4)	
RI	3 (1)	3 (1)	0 (0)	
Infarct location, *n* (%)				**0.019**
Non-anterior	238 (54)	220 (55)	18 (37)	
Anterior	207 (46)	177 (45)	30 (63)	
Number of affected vessels, *n* (%)				**0.002**
1	271 (61)	248 (62)	23 (48)	
2	127 (28)	114 (29)	13 (27)	
3	47 (11)	35 (9)	12 (25)	
TIMI flow pre-pPCI, *n* (%)				0.277
0	288 (65)	252 (63)	36 (75)	
1	64 (14)	58 (15)	6 (12.5)	
2	74 (17)	68 (17)	6 (12.5)	
3	19 (4)	19 (5)	0 (0)	
TIMI flow post-pPCI, *n* (%)				**0.002**
0	8 (2)	7 (2)	1 (2)	
1	6 (1)	3 (1)	3 (6)	
2	46 (10)	37 (9)	9 (19)	
3	385 (87)	350 (88)	35 (73)	
Peak hs-cTnT, ng/l	4925 [2456–8564]	4666 [2188–8015]	7438 [4278–14751]	** < 0.001**

Bold values denote statistical significance

*MACE*  major adverse cardiac events, *RCA*  right coronary artery, *LAD* left anterior descending artery, *LCX* left circumflex artery, *RI* Ramus intermedius, *TIMI*  thrombolysis in myocardial infarction, *pPCI* primary percutaneous coronary intervention, *Hs-cTnT* high-sensitivity cardiac troponin T

### CMR parameters

CMR scans were performed at a median of 3 [IQR: 2–4] days after pPCI. Time to CMR did not differ between patients with and without MACE (*p* = 0.333). Table 2 provides an overview of the assessed CMR parameters, separately for patients with and without MACE.

**Table 2:** CMR results

	Total population (*n* = 445)	No MACE (*n* = 397, 89%)	MACE (*n* = 48, 11%)	*p*-value
LVEDV, ml	152 [127–174]	151 [127–174]	160 [131–174]	0.350
LVESV, ml	72 [55–91]	72 [55–88]	80 [60–107]	**0.041**
LVEF, %	53 [45–59]	53 [45–59]	50 [40–55]	**0.023**
GLS, %	− 12.0 [− 14.0 to − 10.0]	− 12.3 [− 14.1 to − 10.2]	− 10.6 [− 12.4 to − 7.7]	** < 0.001**
GRS, %	27.3 [21.5–32.3]	27.9 [21.7–32.6]	24.6 [18.8–29.8]	**0.030**
GCS, %	− 13.8 [− 15.5 to − 11.6]	− 14.0 [− 15.6 to − 11.7]	− 12.2 [− 13.7 to − 9.3]	** < 0.001**
MAPSE, mm	9.0 [7.4–10.9]	9.1 [7.5–11.0]	8.0 [6.1–9.3]	** < 0.001**
LAS, %	− 14.4 [− 19.6 to − 10.0]	− 15.2 [− 20.0 to − 10.6]	− 11.2 [− 14.9 to − 7.2]	** < 0.001**
IS, % of LVMM	14.0 [7.3–23.7]	13.9 [7.0–23.5]	17.8 [8.8–25.7]	0.095
MVO, *n* (%)	228 (51)	196 (49)	32 (67)	**0.024**
MVO, % of LVMM	0.1 [0.0–1.9]	0.0 [0.0–1.8]	0.9 [0.0–4.4]	**0.031**

Bold values denote statistical significance

*LVEDV*  left ventricular end-diastolic volume, *LVESV* left ventricular end-systolic volume, *LVEF* left ventricular ejection fraction, *GLS* global longitudinal strain, *GRS* global radial strain, *GCS* global circumferential strain, *MAPSE* mitral annular plane systolic excursion, *LAS* fast manual long-axis strain, *IS* infarct size, *LVMM* left ventricular myocardial mass, *MVO* microvascular obstruction

LVEF of the overall population was 53 [IQR: 45–59]%, MAPSE 9.0 [IQR: 7.4–10.9] mm, LAS − 14.4 [IQR: − 19.6 to − 10.0]% and GLS − 12.0 [IQR: − 14.0 to − 10.0]%. Infarct characteristics were as follows: IS 14.0 [IQR: 7.3–23.7]% and MVO 0.1 [0.0–1.9]% of LVMM. MVO occurred in 51% (*n* = 228) of the overall cohort.

### Clinical outcome

During a median follow-up time of 16 [IQR: 12–49] months, 48 patients (11%) suffered from MACE (12 deaths, 17 re-infarctions and 19 heart failure events). Patients with MACE were significantly older (*p* = 0.023) and presented more often with cardiovascular risk factors (diabetes *p* = 0.012 and hypertension *p* < 0.001). The number of diseased vessels was higher (*p* = 0.002) and thrombolysis in myocardial infarction flow post-pPCI was lower in the group with MACE (*p* = 0.002). Furthermore, peak hs-cTnT values were higher in patients with MACE (*p* < 0.001).

### CMR measures and major adverse cardiovascular events

LVEF was significantly lower in patients with MACE (*p* = 0.023). All three LV myocardial strain parameters (GLS, GRS and GCS) were significantly related to MACE (all *p* < 0.05). Accordingly, GLS was higher in the MACE group (− 10.6% vs. − 12.3%, *p* < 0.001). MAPSE was significantly lower in patients with MACE (8.0 mm vs. 9.1 mm, *p* < 0.001), whereas LAS was higher in the MACE group (− 11.2% vs. − 15.2%, *p* < 0.001). Among parameters of myocardial damage, the presence (*p* = 0.024) and the extent of MVO (*p* = 0.031) were associated with MACE (Table 2).

**Fig. 3:**
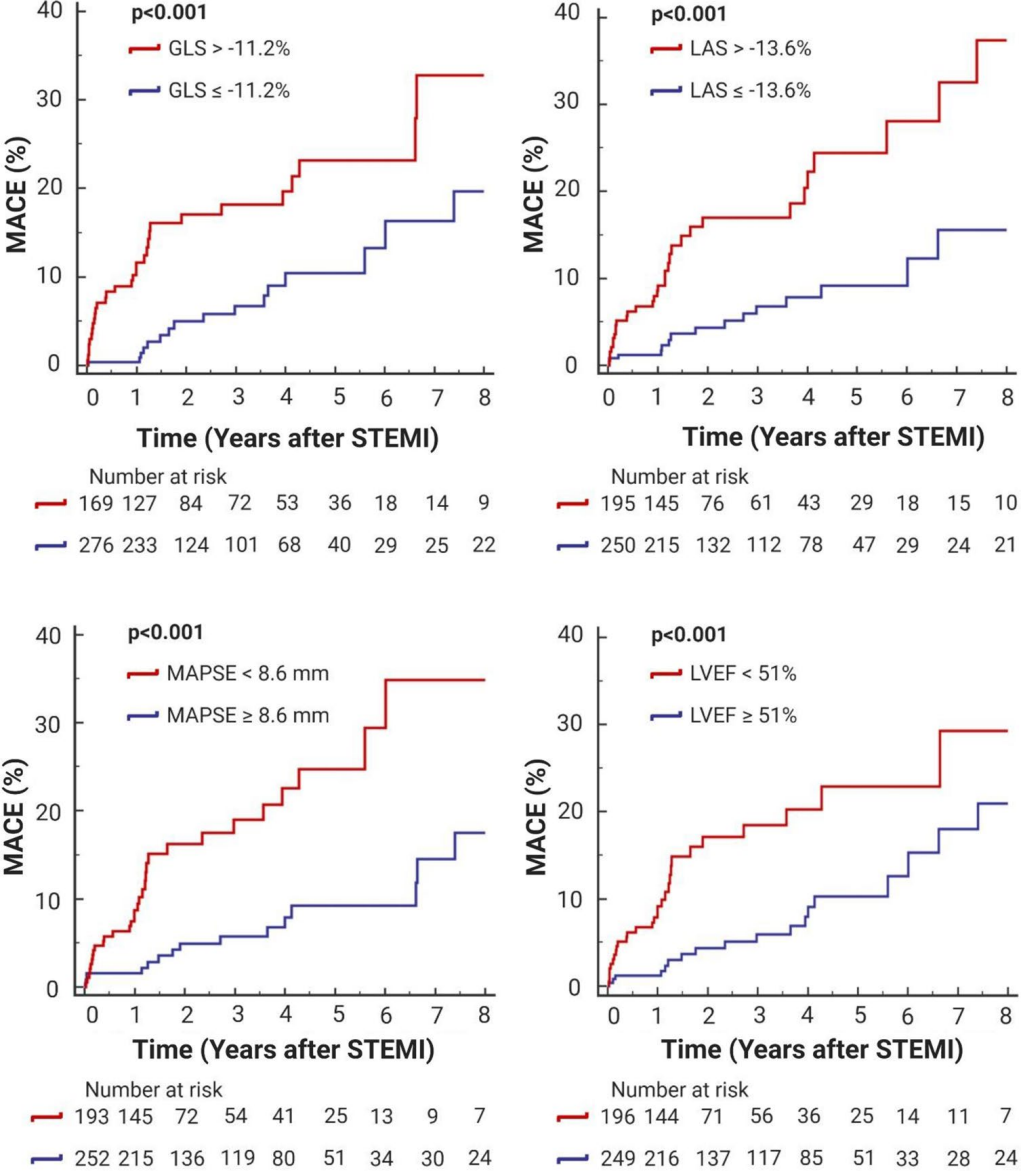
LV functional parameters and clinical outcome. Kaplan–Meier curves displaying event-free survival according to best cut-offs of GLS, LAS, MAPSE and LVEF. *MACE *major adverse cardiac events, *LVEF* left ventricular ejection fraction, *GLS* global longitudinal strain, *MAPSE* mitral annular plane systolic excursion, *LAS* fast manual long-axis strain, *STEMI* ST-segment elevation myocardial infarction

As illustrated by the Kaplan–Meier curves (Fig. 3), GLS > − 11.2%, MAPSE < 8.6 mm, LAS > − 13.5% and LVEF < 51% were associated with a significantly lower MACE-free survival (all *p* < 0.001).

**Fig. 4:**
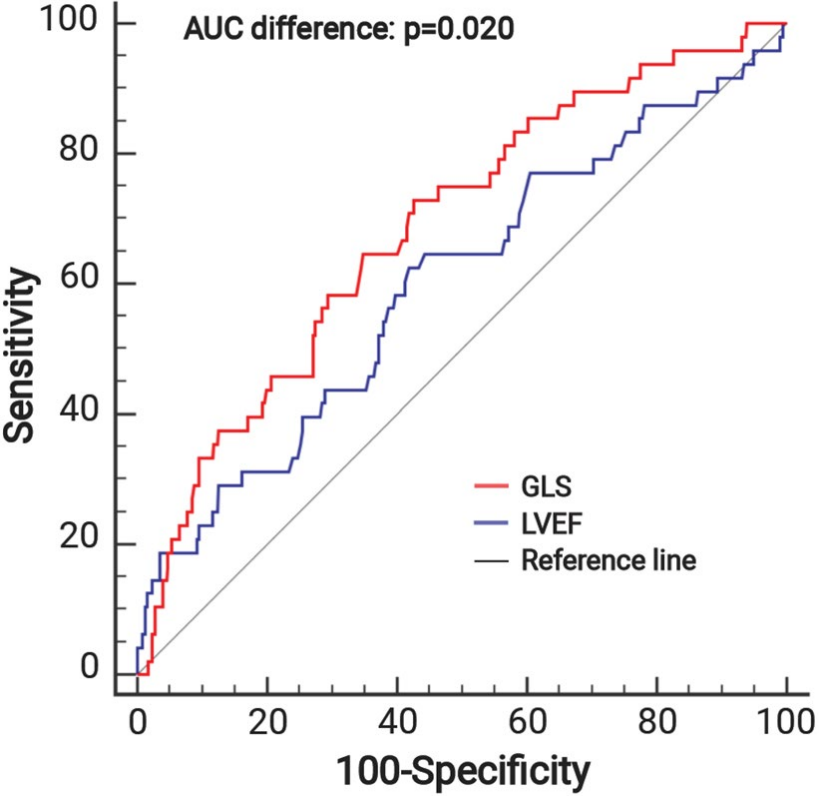
Discriminative prognostic power of GLS and LVEF. ROC curves of GLS in comparison to LVEF for the prediction of MACE. GLS revealed a significantly higher AUC than LVEF (0.69, 95% CI: 0.64–0.73, *p* < 0.001 vs. 0.60, 95% CI: 0.55–0.65, *p* = 0.031. AUC difference: 0.09, *p* = 0.020). *ROC* receiver operating characteristic, *AUC* area under the curve, *MACE *major adverse cardiac events, *LVEF* left ventricular ejection fraction, *GLS* global longitudinal strain,

GLS showed the highest AUC for the prediction of MACE (0.69, 95% CI: 0.64–0.73; *p* < 0.001). In comparison to all investigated LV functional parameters, only the AUC of GLS was significantly higher than the AUC of LVEF (0.60, 95%CI: 0.55–0.65; *p* = 0.031. AUC difference: 0.09; *p* = 0.020, Table 3, Model 1) (Fig. 4).

**Table 3:** C-statistics for the prediction of MACE

Variables	AUC	95% CI	*p*-value	AUC increment	ROC comparison
*Model 1*					
LVEF, %	0.60	0.55–0.65	**0.031**	–	–
LV GLS, %	0.69	0.64–0.73	** < 0.001**	0.09	**0.020**
*Model 2*					
LVEF, %	0.60	0.55–0.65	**0.031**	–	–
LAS, %	0.67	0.62–0.70	** < 0.001**	0.07	0.164
*Model 3*					
LVEF, %	0.60	0.55–0.65	**0.031**	-	-
MAPSE, mm	0.66	0.61–0.70	** < 0.001**	0.06	0.246

Bold values denote statistical significance

*AUC* area under the curve, *ROC* receiver operating curve, *MACE* major adverse cardiac events,                    *LVEF* left ventricular ejection fraction, *GLS* global longitudinal strain, *MAPSE* mitral annular plane systolic excursion, *LAS* fast manual long-axis strain

In reclassification analysis, the addition of GLS to LVEF (continuous net reclassification improvement (NRI): 0.60 [95% CI: 0.31–0.88], *p* < 0.001; IDI: 0.02 [95% CI: 0.01–0.04], *p* < 0.001), LAS to LVEF (continuous NRI: 0.51 [95% CI: 0.23–0.79], *p* < 0.001; IDI: 0.02 [95% CI: 0.01–0.03], *p* = 0.005) and MAPSE to LVEF (continuous NRI: 0.52 [95% CI: 0.24–0.81], *p* < 0.001; IDI: 0.02 [95% CI: 0.01–0.03], *p* = 0.005) led to a significant improvement in risk classification.

In a multivariable CMR model (Table 4) including LVEF, MAPSE, GLS, LAS, extent of MVO and IS, only GLS (HR: 1.13, 95% CI: 1.01–1.27, *p* = 0.030) and LAS (HR: 1.07, 95% CI: 1.01–1.14, *p* = 0.019) significantly predicted MACE. As demonstrated by the clinical model (Table 4), the association between GLS and MACE remained significant (*p* = 0.003) after adjustment for clinical determinants of MACE (age, hypertension, diabetes, number of diseased vessels, peak hs-cTnT). All variables included in multivariable analysis showed a VIF of < 3 (CMR model: 1.3–2.2, angiographical model: 1.1–1.9, clinical model: 1.1–1.6).

**Table 4:** Predictors of MACE in univariable and multivariable Cox regression analysis

	Univariable		Multivariable	
	HR (95%CI)	*p*-value	HR (95%CI)	*p*-value
*CMR model*				
LV EF, %	0.95 (0.93–0.98)	** < 0.001**		
MAPSE, mm	0.80 (0.72–0.89)	** < 0.001**		
LV GLS, %	1.22 (1.12–1.34)	** < 0.001**	1.13 (1.01–1.27)	**0.030**
LAS, %	1.11 (1.06–1.17)	** < 0.001**	1.07 (1.01–1.14)	**0.019**
MVO, % of LVMM	1.13 (1.06–1.22)	**0.001**		
IS, % of LVMM	1.03 (1.01–1.06)	**0.014**		
*Angiographical model*				
LV GLS, %	1.22 (1.12–1.34)	** < 0.001**	1.13 (1.01–1.28)	**0.037**
LAS, %	1.11 (1.06–1.17)	** < 0.001**		
Infarct location	2.17 (1.21–3.89)	**0.010**		
Number of affected vessels	1.63 (1.12–2.38)	**0.011**	1.58 (1.09–2.29)	**0.015**
TIMI flow post-pPCI	0.77 (0.54–1.08)	0.127		
*Clinical model*				
LV GLS, %	1.22 (1.12–1.34)	** < 0.001**	1.16 (1.05–1.29)	**0.003**
Age	1.03 (1.01–1.06)	**0.014**	1.03 (1.00–1.06)	**0.044**
Hypertension	2.95 (1.47–5.94)	**0.002**	2.4 (1.18–4.90)	**0.016**
Diabetes mellitus	2.92 (1.40–6.07)	**0.004**	2.27 (1.08–4.80)	**0.031**
Peak hs-cTnT, ng/l	1.00 (1.00–1.00)	** < 0.001**	1.00 (1.00–1.00)	**0.005**

Bold values denote statistical significance

*HR* hazard ratio, *CI* confidence interval, *MACE*  major adverse cardiac events, *TIMI*  thrombolysis in myocardial infarction, *pPCI* primary percutaneous coronary intervention, *Hs-cTnT* high-sensitivity cardiac troponin T,     *LVEF* left ventricular ejection fraction, *GLS* global longitudinal strain, *MAPSE* mitral annular plane systolic excursion, *LAS* fast manual long-axis strain, *IS* infarct size, *LVMM* left ventricular myocardial mass, *MVO* microvascular obstruction

## Discussion

To the best of our knowledge, this is the first comprehensive study investigating the comparative prognostic value of all currently available functional CMR parameters including LVEF, MAPSE, GLS and LAS in acute STEMI patients revascularized by pPCI. The major findings can be summarized as follows: LVEF, MAPSE, GLS and LAS were significantly associated with MACE. The prognostic value of MAPSE, GLS and LAS was incremental to LVEF. GLS emerged as independent predictor of MACE after adjustment for measures of LV function (LVEF, MAPSE, LAS), myocardial injury (IS and MVO) angiographical (number of diseased vessels, infarct location) and clinical parameters (age, hypertension, diabetes, peak hs-cTnT) with superior prognostic validity compared to LVEF. Taken together, feature tracking of GLS using CMR independently enables risk assessment after STEMI and expands the prognostic significance of LVEF.

### Prognostic value and timing of imaging

As recommended by the current guidelines, myocardial function should be determined in all patients with acute STEMI before hospital discharge [2]. Due to its broad and easy availability, LVEF by echocardiography is usually the preferred LV functional parameter for risk stratification after STEMI in daily clinical routine [2]. Over the last years, analyses of myocardial strain and MAPSE have primarily been driven by echocardiography; however, echocardiographic techniques are limited by moderate spatial resolution and often hampered by an insufficient acoustic window [24, 25]. Indeed, the assessment of myocardial function by CMR has increasingly focused on risk stratification after STEMI [26, 27]. Due to its high spatial and temporal resolution, CMR enables accurate assessment of GLS by tracking the endo- and epicardial borders precisely. These semi-automatic post-processing mechanisms allow quick and easy analysis of LV deformation parameters [15]. On the other hand, echocardiography is a broadly available, cost-effective imaging tool, but more susceptible to poor image quality [24]. This could be due to the examiner as well as the person to be examined complicating the exact assessment of these parameters [24]. Even though CMR accurately depicts myocardial alterations, local experience might influence the use of CMR vs. echocardiography-guided risk assessment strategies.

In a previous work, we revealed CMR assessed MAPSE to independently predict the occurrence of hard clinical events with superior prognostic validity than LVEF following acute STEMI [9]. However, the current work expands our previous findings by highlighting MAPSE to be significantly associated with MACE incremental to LVEF in an even larger cohort of STEMI patients. As a great advantage, MAPSE can be quickly evaluated on cine CMR images without the need of a special post-processing software.

LAS is another parameter of global systolic function which can be easily assessed from standard cine CMR images [8] without proprietary software. Schuster and colleagues demonstrated in a large cohort of patients with acute myocardial infarction a strong association of impaired LAS with MACE occurrence. In their study, GLS was also found to be a significant predictor of MACE if calculated separately from LAS. However, it was not included in multivariable analysis due to its high correlation with LAS as stated by the authors. On the basis of the VIF, we could include all LV functional parameters in multivariable analysis and could demonstrate GLS and LAS to independently predict MACE in the CMR model. However, GLS, but not LAS, remained an independent predictor of MACE following acute STEMI after adjustment for clinical parameters. Since LAS assesses the relative distances between mitral annular and apical planes in long-axis views, it is unable to provide regional myocardial information which might be of major prognostic relevance in the context of acute myocardial infarction [8].

Feature tracking CMR is a high-resolution investigation of global and regional myocardial deformation by tracking the actual myocardial borders and following them subsequently [15, 28]. Depicting the 3-dimensional LV deformation during the cardiac cycle is challenging due to the complex interplay between longitudinal and circumferential shortening, radial thickening and torsion [29]. Especially longitudinal myocardial fibers seem to be sensitive to ischemia, possibly due to their subendocardial location [11]. However, there is an ongoing debate whether myocardial fiber architecture has an impact on cardiac function after myocardial infarction. Our research group recently demonstrated feature tracking CMR gained GLS to strongly and independently predict the occurrence of MACE in the medium term after acute STEMI [14]. This fits well with other CMR studies [15, 30] supporting the findings of our present study. Finally, we could confirm and expand previous results [14, 15, 30] by showing that GLS remained a significant predictor of MACE even after adjustment for IS and MVO, which are considered as major determinants of worse prognosis following STEMI [31], angiographical and clinical parameters. Interestingly, Backhaus and colleagues revealed that infarct-related artery alterations after myocardial infarction might have an impact on subsequent prognosis [32]. In their analysis, patients with left circumflex as infarct-related artery, GLS best predicted MACE, whereas in left anterior descending artery and right coronary artery infarcts, left atrial total strain was more expressive for prognosis assessment. This underlines the complexity of pathophysiological mechanisms leading to myocardial deformation.

As emphasized previously, the optimal timing for measurement of myocardial deformation is unclear [33]. In accordance with other CMR studies investigating the prognostic value of LV functional parameters [8, 9, 14, 15, 30], we performed CMR imaging within a week after STEMI. However, in contrast to Schuster et al. [8], time to CMR in our cohort did not differ between patients with and without MACE. This might be explained by the fact, that we only included hemodynamic stable STEMI patients (Killip < 3) which were able to undergo CMR investigations within the proposed time range. Future studies in this direction are needed to validate the optimal time point for myocardial deformation measurement with CMR after STEMI.

### Clinical implications and future directions

We could demonstrate that GLS as a single marker independently improved personalized risk stratification after STEMI with superior prognostic validity compared to LVEF. On the other hand, we demonstrated that the predictive value of LAS or MAPSE in addition to LVEF was higher than that of LVEF alone. These combinations might serve as good alternatives for LV-based risk assessment after STEMI, if GLS is not available. As advantages, LAS and MAPSE are faster to measure and require no proprietary software compared to GLS [33]. However, additional time and software required for GLS assessment are considered worthwhile since prognostic information beyond established LV functional parameters is improved [34]. GLS is considered as more sensitive marker for LV function-based prognosis assessment [15] and might be impaired despite preserved LVEF as we demonstrated in our cohort. In the current era of pPCI, a relevant portion of STEMI patients presents with a near normal or even preserved LVEF and would therefore be considered as low-risk patients [35]. Nevertheless, the absolute number of MACE is substantial in this subgroup with preserved LVEF [36]. This corroborates the limited prognostic validity of LVEF and advances the need for novel risk stratification tools in STEMI patients with preserved LVEF. Recently, GLS has increasingly come into focus not only for risk stratification but also for representing a potential new therapeutic target [34]. In fact, the METOCARD-CNIC trial by Podlesnikar et al. revealed that early intravenous administration of metoprolol in anterior STEMI patients was accompanied by improved LV strain at 1 week and with fewer patients having altered strain at 1 week and at 6 months than the control group [37]. However, the benefit of anti-remodeling treatment strategies, especially in patients with preserved LVEF (but maybe altered strain values) has not been clarified so far. Finally, the manner in which imaging-driven strategies of myocardial deformation assessment will support daily clinical decision-making requires further research [33, 38].

### Limitations

In this observational CMR study, only stable patients (Killip class < 3) with acute and first-time STEMI were included; hence, our findings may not be transferable to hemodynamically less stable patients or to patients with previous myocardial infarction. Notably, the vast majority of STEMI patients presents with Killip class < 3 [39]. Although, quantitative native T1 mapping and post-contrast T1 mapping (including extracellular volume), as well as T2* mapping, were recently suggested for improved risk stratification following STEMI [40–43], we could not include these measurements due to unavailability of these sequences for a considerable number of patients. Furthermore, CMR investigations were conducted at a single time point and within a week after the index event for LV deformation measurements. Defining the optimal time frame for performing CMR remains another important area for future research. Even though CMR is the optimal imaging tool for quantification of LV parameters [4], it exhibits limitations, such as restricted availability, higher costs and center-specific expertise. Furthermore, LGE CMR has some contraindications, such as non-magnetic resonance conditional pacemakers or other ferromagnetic structures and implants, renal function impairment and claustrophobia. In contrast, echocardiography is widely available, has significantly lower costs, is faster in image acquisition and can be used “bed-side”. However, echocardiography is highly examiner-dependent and limited acoustic windows may complicate accurate quantification of LV deformation [3, 25]. Further studies are needed to investigate the comparative predictive value of all LV deformation parameters as assessed by echocardiography and CMR. The lack of a validation cohort and the relatively small number of events in our cohort represent an important limitation of the study. Finally, inconsistencies between commercially available software for strain assessment with acceptable differences for GLS and GCS, but not for GRS have to be taken into account [44].

## Conclusion

GLS by feature tracking CMR emerged as independent predictor of MACE with superior prognostic validity compared to LVEF. The combination of LAS or MAPSE with LVEF revealed a significantly better prognostic value than LVEF alone and could be used as alternatives for risk stratification after STEMI.

## Abbreviations

AUC: Area under the curve
CMR: Cardiac magnetic resonance
ECG: Electrocardiographic
FPS: Frames per second
FA: Flip angle
FOV: Field of view
GCS: Global circumferential strain
GLS: Global longitudinal strain
GRS: Global radial strain
Hs-cTnT: High sensitivity cardiac troponin T
IDI: Integrated discrimination improvement
IQR: Interquartile range
IS: Infarct size
LAS: Fast manual long-axis strain
LGE: Late gadolinium enhancement
LV: Left ventricular
LVEF: Left ventricular ejection fraction
LVMM: Left ventricular myocardial mass
MACE: Major adverse cardiac events
MAPSE: Mitral annular plane systolic excursion
MVO: Microvascular obstruction
NRI: Net reclassification improvement
pPCI: Primary percutaneous coronary intervention
ROC: Receiver operating characteristic
SL: Slice thickness
STEMI: ST-segment elevation myocardial infarction
TE: Echo time
TR: Repetition time
VIF: Variance inflation factor
VS: Voxel size
